# Case Report: Gene expression profiling of COVID-19 vaccination-related lymphadenopathies reveals evidence of a dominantly extrafollicular immune response

**DOI:** 10.3389/fimmu.2023.1285168

**Published:** 2023-11-14

**Authors:** Thomas Menter, Carl P. Zinner, Christoph T. Berger, Philip Went, Alexandar Tzankov

**Affiliations:** ^1^ Pathology, Institute of Medical Genetics and Pathology, University Hospital Basel, University of Basel, Basel, Switzerland; ^2^ University Center for Immunology, University Hospital Basel, Basel, Switzerland; ^3^ Department Biomedicine, Translational Immunology, University of Basel, Basel, Switzerland; ^4^ Institute of Pathology, Cantonal Hospital Chur, Chur, Switzerland

**Keywords:** gene expression profiling, COVID-19, TLR, lymphadenopathy, plasmablast, vaccine, mRNA

## Abstract

mRNA-based vaccines against SARS-CoV-2 have been proven to be very efficient in preventing severe COVID-19. Temporary lymphadenopathy (LA) has been observed as a common adverse event following immunization. Here we describe a case series of three female patients with prominent local to generalized LA after SARS-CoV-2 mRNA-1273 vaccination, which led to lymph node biopsy due to the suspicion of lymphoma or metastasis. All three patients morphologically showed similar patterns of follicular hyperplasia and especially extrafollicular blast activation. Two of the three patients only had short-lasting humoral immune responses to the vaccination. Gene expression profiling (GEP) using the HTG *Immune response panel* revealed that all three patients clustered together and clearly differed from the GEP-patterns of COVID-19, infectious mononucleosis and non-specific follicular hyperplasia. The closest similarities were seen with lymph nodes showing extrafollicular activation of B-blasts as well as hemophagocytosis. The GEP of the vaccination-induced LA was reminiscent of an immune response with little potential of immunologic memory. mRNA-1273 vaccination-induced LA may to a certain extend reflect disordered immune response with potentially poor immunologic memory in affected individuals.

## Introduction

Lymphadenopathy occurring in the wake of vaccination is a well-recognized phenomenon described in a plethora of vaccines administered (1, 2). Its morphology ranges from the presence of granulomas (especially in correlation with bacillus Calmette-Guérin, BCG) to florid hyperplasia and non-specific enlargement of lymph nodes.

mRNA-based vaccinations have been proven to be very successful in taming the COVID-19 global pandemic (3). Since their release, more than 13 billion doses of this new immunization type have been administered worldwide (4). In the phase III studies of the mRNA COVID vaccines, lymphadenopathy, mainly restricted to axillary lymph nodes at the site of injection and limited to <10 days, occurred in 0.3% (BNT162b) and <0.1% (mRNA-1273) as a potential adverse event following immunization (AEFI) (5, 6). Local and short-lasting lymphadenopathy is typically considered a manifestation of the evolving immune response to immunization, but lymphadenopathy may also persist over weeks and/or be generalized. These findings are subsumed by the term “COVID-19 vaccine-associated lymphadenopathy”, which was the most common (8 in 10’000 vaccinated subjects) AEFI of interest in a large cohort study of vaccinated individuals (7).

Especially in patients who are suffering or have previously been suffering from cancer, the rapid onset of lymphadenopathy raises the suspicion of progressive disease. Furthermore, lymphoma is a potential differential diagnosis in symptomatic patients with lymphadenopathy that is not confined to the injection-site draining region. Notably, clonally restricted plasma cells have been reported in the setting of COVID-19 vaccine-associated lymphadenopathy (8). Several studies described the morphology of these lymph nodes,, and many studies showed rather non-specific results (9). Our own observations (10) revealed a rather recurrent pattern of extrafollicular proliferation of B-blasts that reflects a rapid B-cell-expansion as primary antigen-reaction that bypasses the germinal center reaction (11).

In this case series, we present three women with COVID-19 vaccine-associated lymphadenopathy including a profound description of both morphologic changes of the lymph nodes and provide unprecedented comprehensive gene expression profiling in comparison to various lymphadenopathies including COVID-19.

## Methods

### Patient cohort and study design

A total of three original cases of mRNA-1273 vaccination-induced lymphadenopathies and 52 reference lymph node samples were examined. These control cases have been recently reported (12) and included draining pulmonary lymph nodes of 25 lethal COVID-19, 6 unremarkable (mediastinal) lymph nodes, 6 peripheral lymph nodes with follicular hyperplasia, 6 infectious mononucleosis lymphadenopathies, 5 lymphadenopathies accompanying hemophagocytic lymphohistiocytosis and 4 lymph nodes with extrafollicular plasmablast activation. Autopsies of lethal COVID-19 were performed along the first large scale report in the pandemics (13–15). This study has received approval by the Ethics Committee of Northwestern and Central Switzerland (ID 2020-00629).

### Gene expression profiling and cell type deconvolution by cibersortx

GEP was performed by HTG according to established protocols using the Immune Response Panel. The approximate composition of immune cell types was inferred from the bulk-RNA data utilizing the cibersortx web tool. Further details on sample processing and data evaluation in these respects are provided in the supplementary file (**Supplementary Material**).

## Results

### Case presentations

Laboratory values of our patients at the time of biopsy are provided in **Supplementary Table 1**.

**Figure 1:**
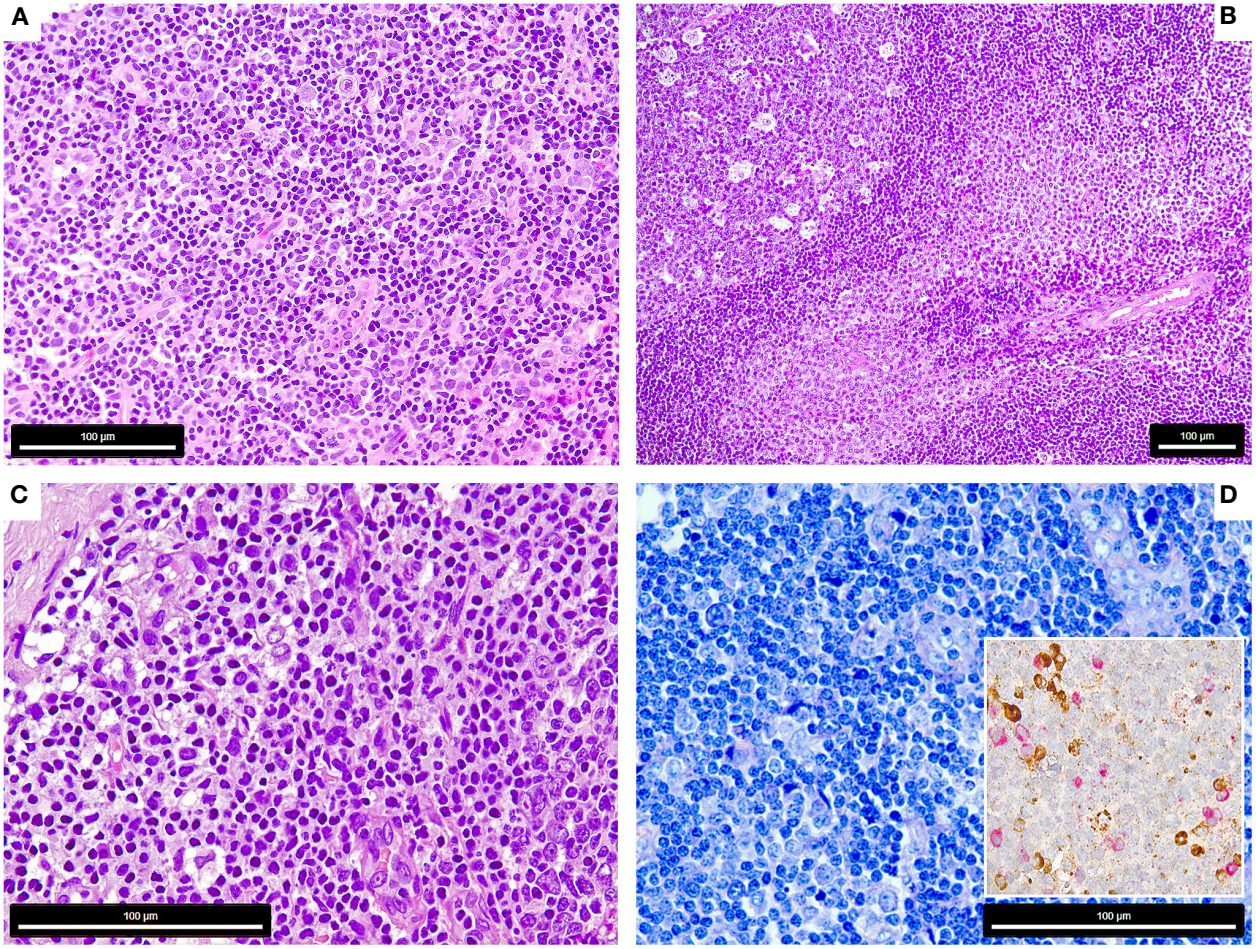
Morphology of the COVID-19 vaccination-associated lymphadenopathies **(A)** Patient 1, showing paracortical hyperplasia and prominence of extrafollicular B-blasts and plasmablasts (H&E, 400x); **(B)** Patient 2, showing follicular (left) and moncytoid B-cell hyperplasia (right) and presence of extrafollicular B-blasts (bottom right) (H&E, 200x); **(C, D)** Patient 3, showing extrafollicular B-blasts and plasmablasts, which are polytypic with respect to light chain expression (insert) [**C**: H&E, 600x, **D**: Giemsa 600x, insert: immunohistochemistry for Kappa (brown) and Lambda (red) light chains, 650x].

*Case 1* is a 73-year-old Caucasian woman with a previous history (3 years ago) of a limited-stage (pT1b, pN0, cM0, R0) cancer of the left breast that has been treated with resection, local radiotherapy including the left axilla and anti-hormonal therapy with letrozole continuing at that time. In addition, she suffered from rheumatoid arthritis treated with leflunomide. The patient has not reported a SARS-CoV2-infection prior to her vaccination. She presented with massively enlarged left-axially lymph nodes at the side of the vaccination 8 days after the first dose of the COVID-19 mRNA-1273 vaccine. She did not complain of other side effects. The axillary lymph nodes were biopsied and showed an increase of polyclonal plasma cells and extrafollicular proliferation of B-blasts/plasmablasts (**Figure 1A**). No further imaging studies had been performed along to the biopsy. Based on the diagnosis of AEFI, she has been recommended to proceed for as per protocol recall vaccination in a regional (cantonal) hospital in Switzerland but was lost from follow-up and her anti-SARS-CoV-2 S-antigen-antibody titers were therefore not available.

*Case 2* is a 44-year-old Caucasian woman, who suffered from rapid onset fatigue, splenomegaly and tonsillitis, which retrospectively was diagnosed as infectious mononucleosis based on corresponding serology findings for Epstein Barr virus (EBV) (16). Four weeks later, she received the first dose of COVID-19 mRNA-1273 vaccination, followed by prominent adverse events including supraclavicular lymphadenopathy, fever, night sweats and significant weight loss. As symptoms became even more pronounced after receiving the second dose of mRNA-1273 and she developed a bilateral cervical lymphadenopathy, a supraclavicular lymph node excised in a regional (cantonal) hospital in Switzerland showed florid follicular and paracortical hyperplasia with excess of monocytoid B-cells, extrafollicular B-blasts and an increase of plasmacytoid dendritic cells (**Figure 1B**), but no evidence of EBV as assessed by *in situ* hybridization for EBV small RNA. Also this patient has not reported a SARS-CoV2-infection prior to her vaccination. After 8 months, anti-SARS-CoV-2 S-antigen antibody levels decreased significantly, but still remained above the targeted threshold (16). When the patient received her booster vaccination (half dose of BNT162b; switch due to the previous AEFI) another 9 months later, she experienced no recurrence of lymphadenopathy, fatigue or fever and her anti-S-antigen antibody titers increased again as measured on three months of follow-up.

*Case 3* is a 66-year-old Caucasian woman who presented with high fever, skin rash and myalgia after the second COVID-19 mRNA-1273 vaccination. FDG-PET/CT performed at an university hospital (tertiary care center) in Switzerland showed generalized FDG+ lymphadenopathy and hypermetabolic activity of the spleen (**Supplementary Figure 1**). This patient has neither reported a SARS-CoV2-infection prior to her vaccination, nor was her serology suggestive of previous SARS-CoV2-infection. Most symptoms resolved spontaneously, but fatigue and lymphadenopathy persisted for two months. She underwent a left-axillary lymph node excision to rule out lymphoma. Morphologic examination of this lymph node showed florid follicular hyperplasia and extrafollicular B-blasts (**Figures 1C, D**). In her previous medical history, the patient has been documented with immunologically controlled chronic hepatitis B (HBV)-infection, allergic asthma (both not in need of treatment) and had episodes of Malaria. Four weeks after the second vaccine her anti-S-IgG was >2500 U/l, but decreased below the targeted threshold at 6 months. Similar to case 2, she did not show a relapse of her symptoms when receiving the third dose of mRNA-1273.

### Morphologic assessment

The lymph nodes of all three patients showed signs of activation of the immune system with follicular hyperplasia with a particular expansion of the parafollicular zone with marked increase of extrafollicular B-blasts, augmentation of polyclonal plasma cells and plasmablasts (**Figures 1A–D**). Case 2 also showed a prominent increase of monocytoid B-cells and plasmacytoid dendritic cells (**Figure 1B**) (16).

### Gene expression profiling

**Figure 2:**
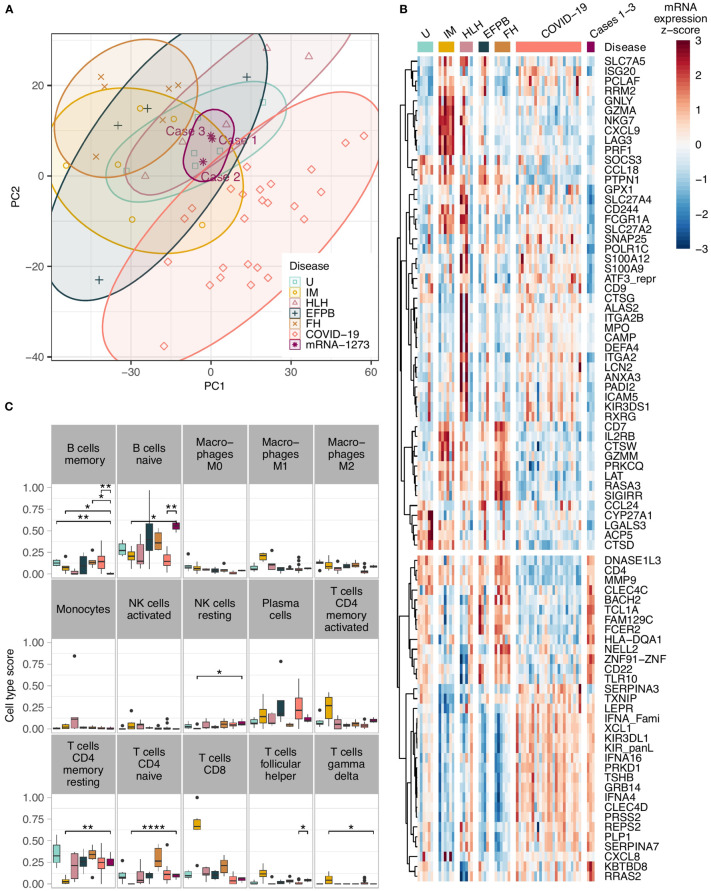
**(A)** Principle component analysis showing a tight clustering of the COVID-19 vaccination-associated lymphadenopathy cases in comparison to other entities; note the cluster overlap of the COVID-19 vaccination-associated lymphadenopathy cases with cases of hemophagocytic lymphohistiocytosis and cases with extrafollicular (plasma-)blast activation; **(B)** Heat-map of the top differentially expressed genes in COVID-19 vaccination-associated lymphadenopathies compared to various other lymphadenopathies. All genes have an absolute fold change >2 and FDR – *P*<0.05 between the vaccination-associated cases and at least one of the control groups. The unsupervised hierarchical clustering of the gene correlations assembles most of the vaccination-associated case downregulated genes on the upper part, and the upregulated genes on the lower part; **(C)** Comparison of the *in silco* deconvolution of the immune cells composition in various lymphadenopathies utilizing the cibersortx algorithm; note the significantly higher gene expression signature of naïve B-cells and lower signature of memory B-cells in COVID-19 vaccination-associated lymphadenopathy (as in the other figures, the vaccination-related lymphadenopathies are shown in deep purple/on the right of the graphs; *P<0.05, **P<0.01, and ****P<0.001 after FDR adjustment). U, unremarkable (mediastinal) lymph nodes (pale green); IM, infectious mononucleosis (gold); HLH, hemophagocytic lymphohistiocytosis (pale purple); EFPB, extrafollicular plasmablast activation (dark green); FH, follicular hyperplasia (pale brown); COVID-19 draining pulmonary lymph nodes of lethal COVID-19 (orange); mRNA-1237 COVID-19 vaccination-associated lymphadenopathy (deep purple).

Applying the Autoimmune Panel also known as Immune Response Panel by HTG Molecular®, all three lymph nodes of COVID-19 mRNA-1273-induced lymphadenopathy clustered closely together in the principle component analysis (**Figure 2A**). They were distinctly separated from mononucleosis lymphadenopathies (except for case 2, in which the patient had a history of infectious mononucleosis preceding by a few weeks the mRNA-1273-induced lymphadenopathy), follicular hyperplasias and draining pulmonary lymph nodes of lethal COVID-19. Most overlaps were seen with cases of hemophagocytic lymphohistiocytosis and cases with extrafollicular (plasma-)blast activation (**Supplementary Figure 2**). Also, when comparing the mRNA-1273-induced lymphadenopathy against all other cases, a large quantity of significant differences regarding the gene expression profile were seen, e.g. upregulation of *SOCS5* and *MAP2K4* but also *BTK*, and downregulation of *IL2RB* and *BAX* but also *CD244* (**Supplementary Figure 3**).

Pathways related to interleukin (IL) 10 and interferon gamma (INF-γ) signaling as well as antigen presentation were downregulated in mRNA-1273-induced lymphadenopathy compared to cases of infectious mononucleosis, while CD20 (*MS4A1*) and *IL2* were upregulated (**Supplementary Figure 2B**, **Supplementary Results**). In contrast to cases of follicular hyperplasia not related to vaccination, pathways related to autophagy, proinflammatory and profibrotic mediators and JAK/STAT signaling were activated in mRNA-1273-induced lymphadenopathy, while IL17- and T-cell dependent pathways and apoptotic signaling as well as *CD44* were downregulated (**Supplementary Figure 2E**). The three patients with mRNA-1273-induced lymphadenopathy showed marked activation of B-cell and T-cell signaling as well as signs of activation of the immune system in general compared to lymph nodes of COVID-19 patients (**Supplementary Figure 2F**). On the opposite, the latter showed activation of pathways related to thromboinflammation and activation of macrophages, whereas pathways involved in complement activation and coagulation were downregulated in mRNA-1273-induced lymphadenopathy. Compared to normal controls (unremarkable mediastinal lymph nodes), proliferation-related genes were clearly upregulated (**Supplementary Figure 2A**); furthermore, macrophages were activated as seen by upregulation of *CD14*, *CD68, LYZ* and *SOCS3*; in contrast, *TCL1A*, which is involved in preventing apoptosis of B-cells, was increased in the vaccination group. The comparison between the vaccination cohort and cases with extrafollicular blast activation did not reveal differences except stronger INF-α signaling in the vaccination group.

Another interesting feature in the vaccination group was a consistent upregulation of the Toll-like receptor (TLR) 10 in the vaccination group compared to infectious mononucleosis, COVID-19 patients and hemophagocytosis (**Figure 2B**); there also was a trend to a general upregulation compared to all controls, which did not yet reach statistical significance (**Supplementary Figure 3**). All other TLRs were not upregulated in the vaccination group.

### Cell type deconvolution by cibersortx

By *in silco* analysis of the composition of the immune cells in the various lymph nodes that was deduced based on quantitation of the respective gene expression, so called naïve B-cells were significantly more frequent in the mRNA-1273-induced lymphadenopathy cohort compared to draining pulmonary lymph nodes of lethal COVID-19, lymph nodes of hemophagocytic lymphohistiocytosis and infectious mononucleosis (**Figure 2C**; **Supplementary Figure 4**). In contrast, memory B-cells were considerably less frequent - actually lowest among all studied cases - in COVID-19 vaccination-associated lymphadenopathy compared to follicular hyperplasia non-related to vaccination. As expected, CD4-positive memory T-cells were less frequent, while M1-macrophages, activated NK-cells and CD8-positive cytotoxic T-cells were more frequent in cases of infectious mononucleosis than in all other lymphadenopathies. M2-macrophages were more abundant in COVID-19 vaccination-associated lymphadenopathy than in COVID-19 patients.

## Discussion

This is the first study evaluating COVID-19 vaccination-associated lymphadenopathy by gene expression profiling and in comparison to other lymphadenopathies. We demonstrate that affected lymph nodes show a prototypic pattern that clusters separately from lethal COVID-19, infectious mononucleosis and follicular hyperplasia of other causes than vaccination but overlaps with extrafollicular activation of B-blasts and hemophagocytosis.

In addition and though based on a few cases, our findings suggest poor immunologic memory in occasions in which vaccination provoked lymphadenopathy by multiple levels of evidence: 1^st^ extrafollicular activation of B-blasts that was the common denominating morphologic pattern observed in the enlarged lymph nodes usually generates short lived plasma cells without immunoglobulin class switch and somatic hypermutation [10], 2^nd^ COVID-19 vaccination-associated lymphadenopathies showed the lowest levels of memory B-cells among all studied lymphadenopathies, 3^rd^ antibody titers against the SARS-CoV-2 S-antigen waned rapidly in the two patients with available follow-up.

So far, most reports and metaanalyses of COVID-19 vaccination-associated lymphadenopathy remained merely descriptive due to sparse and diverse data quality (9, 17). Typically, findings were generically described as follicular hyperplasia or “reactive”, occasionally with the pattern of necrotizing-histiocytic lymphadenopathy as that observable in Kikuchi-Fujimoto-disease (18). Mostly, neither clinical follow-up, gene expression profiling, nor correlation with the humoral immunity induced by immunization was provided. Indeed, the majority of reports have given attention to the differential diagnosis of a malignant process (lymphoma/metastasis) and have not dissected the pathophysiology of the lymphadenopathy itself. In parallel, comprehensive analyses of reactive lymph node conditions are challenging to find in the literature. The here observed morphologic pattern of extrafollicular activation of B-blasts in addition to follicular hyperplasia was first described in the late 1960ies in lymphadenopathies related to smallpox vaccinations (2). Forty years later, Brighenti et al. then characterized these extrafollicularly activated B-blasts in great detail, showing that this pattern is the morphological correlate of a rapid B-cell response circumventing the germinal center reaction (11). The observed overlap of COVID-19 vaccination-associated lymphadenopathy with cases of hemophagocytic lymphohistiocytosis and cases with extrafollicular (plasma-)blast activation, both reflecting innate or primitive immune responses, also supports our assumption that the former is more tightly linked to immunologic dysfunction. Taking also into consideration the lowest levels of memory B-cells in COVID-19 vaccination-associated lymphadenopathies among all studied lymphadenopathies, it can be speculated that this extrafollicular activation of B-blasts leads to a fast and space-consuming, but less effective immune response to antigens in comparison to the classical germinal center reaction without creating memory B-cells, thus likewise explaining the waning antibodies against the SARS-CoV-2 S-antigen observed in two of our patients.

In accordance with the recurrent pattern of extrafollicular activation of B-blasts in the lymph nodes of the studied patients, there were also some remarkable similarities regarding their clinical findings: all three patients were female and all had a history of immune deregulation (breast cancer with adjuvant radio-antihoromonotherapy combined with rheumatoid arthritis that was under leflunomide, recent EBV infection, and history of malaria, asthma and immunologically controlled HBV-infection). A common molecular finding was the upregulation of *TLR10*. TLRs play an essential role in the recognition of pathogens [analysis of pathogen-associated molecular patterns (PAMP) of infectious agents] and, thus, in priming the immune response. In classical immune responses to vaccinations there is increased NF-κB signaling via TLR4, TLR7 and/or TLR9 (19), while mRNA vaccines have been suggested to activate MDA5, RIG-I and to a lesser extent TLR3, TLR7 and TLR9 (20). Opposite to other TLRs, TLR10-mediated signaling does not activate the immune system and shows immunosuppressive effects (21). TLR10 is predominantly expressed on B-cells and monocytes, yet not detected on T-cells (22). A study on the likelihood of osteitis after BCG-vaccination in newborns revealed a protective role of some *TLR10* single nucleotide polymorphisms for preventing this complication (23). Considering the other findings in our cohort, it is well probable that increased TLR10-signaling might have contributed to the observed reduced response to the vaccination. Further experimental studies on the influence of TLR10 on vaccination-induced immunity/immunopathology are needed to further clarify this issue.

The major limitation of our study is of course the small sample size of only three patients included. Considering this, we mainly focused on genes either consistently altered in all three patients and genes closely related to vaccine-induced changes, and those with immunomodulatory effects. We aimed to see this case series as a starting point for further investigations of reactive lymph changes in the wake of SARS-CoV-2-vaccinations since corroboration of our findings might also pave the way for a better understanding of the pathophysiology of vaccination non-responders.

Taken together, based on these three cases, we provide molecular evidence that COVID19-vaccination lymphadenopathy is distinct from COVID-19 lymphadenopathy and may reflect a somewhat dysfunctional immune response of extrafollicular activation of B-blasts that usually generates short-lived plasma cells likely without significant immunologic memory. We could identify a potential genetic mechanism (upregulation of TLR10), which might be linked to the observed findings and might get attention for larger studies on cohorts of vaccine-related lymphadenopathies.

## Data availability statement

The raw data supporting the conclusions of this article will be made available by the authors, without undue reservation.

## Ethics statement

The studies involving humans were approved by Ethics Committee of Northwestern and Central Switzerland. The studies were conducted in accordance with the local legislation and institutional requirements. The participants provided their written informed consent to participate in this study. Written informed consent was obtained from the individual(s) for the publication of any potentially identifiable images or data included in this article.

## Author contributions

TM: Conceptualization, Data curation, Writing – original draft, Writing – review & editing. CZ: Data curation, Formal Analysis, Visualization, Writing – original draft. CB: Writing – review & editing. PW: Writing – review & editing. AT: Conceptualization, Funding acquisition, Methodology, Resources, Supervision, Visualization, Writing – original draft.
