# Supplementary material for: Case Report: Gene expression profiling of COVID-19 vaccination-related lymphadenopathies reveals evidence of a dominantly extrafollicular immune response
Source: Front Immunol. 2023 Nov 14;14:1285168. doi: 10.3389/fimmu.2023.1285168 (PMC10682704; doi:10.3389/fimmu.2023.1285168)
Supplement: Supplementary file 1 [file Table_1.docx]

**Suppl. Table XY.** Laboratory values of the patients at the time of biopsy.

|  | **Patient 1** | **Patient 3** | **Patient 2** |
| --- | --- | --- | --- |
| **Clinical chemistry** | | | |
| Na (mmol/l) | 140 | 139 | 142 |
| K (mmol/l) | 3.6 | 4.5 | 4.8 |
| Creatinine (μmol/l) | 72 | 61 | 58 |
| GFR (CKD-EPI) (ml/min/1.73m^2^) | 72 | 91 | 104 |
| CRP (mg/l) | 1.5 | **23.2 ↑** | 7.9 |
|  | | | |
| **Blood counts** | | | |
| Leukocytes (10^3^/μl) | 6.9 | 6.42 | 7.1 |
| Erythrocytes (10^6^/μl) | 4.18 | **4.13 ↓** | 4.82 |
| Hemoglobin (g/l) | 123 | **116 ↓** | 136 |
| Hematokrit (%) | 36 | **0.34 ↓** | 42 |
| MCV (fl) | 87 | 82 | 87 |
| MCH (pg) | 29 | 28.2 | 28.2 |
| MCHC (g/l) | 340 | 342 | 324 |
| Thrombocytes (10^3^/μl) | 253 | 261 | 293 |
|  | | | |
| Immature myeloid cells (% / 10^3^/μl) | 0.1/0.01 | 1.3/0.08 | 1.2/0.09 |
| Neutrophils (% / 10^3^/μl) | 63.0/4.32 | 46.4/2.98 | 37.5/2.66 |
| Eosinophils (% / 10^3^/μl) | 1.6/0.11 | **8.2/0.53 ↑** | 0.6/0.04 |
| Basophils (% / 10^3^/μl) | 0.3/0.02 | 1.4/0.09 | 1/0.07 |
| Monocytes (% / 10^3^/μl) | 6.6/0.45 | 4.1/0.26 | 6.8/0.48 |
| Lymphocytes (% / 10^3^/μl) | 28.5/1.95 | 38.6/2.48 | **52.9/3.76** **↑** |

Values beyond the normal range are marked in bold.
