# Supplementary material for: Case Report: Gene expression profiling of COVID-19 vaccination-related lymphadenopathies reveals evidence of a dominantly extrafollicular immune response
Source: Front Immunol. 2023 Nov 14;14:1285168. doi: 10.3389/fimmu.2023.1285168 (PMC10682704; doi:10.3389/fimmu.2023.1285168)
Supplement: Supplementary file 2 [file DataSheet_1.docx]

**Gene expression profiling of COVID-19 vaccination-related lymphadenopathies reveals evidence of a dominantly extrafollicular immune response**

**Supplementary material and supplementary figure legends**

Thomas Menter^1^, Carl P. Zinner^1^, Christoph T. Berger^3^, Philip Went^2^, Alexandar Tzankov^1^

^1^ Pathology, Institute of Medical Genetics and Pathology, University Hospital Basel,

University of Basel, Switzerland

^2^ Institute of Pathology, Cantonal Hospital Chur, Switzerland

^3^ Medical Outpatient Clinic and Translational Immunology, Department Biomedicine, University Hospital Basel, Switzerland.

**Histologic samples used for data accrual**

For gene expression profiling and cell type deconvolution, whole slides of lymph node biopsies were analyzed. All different compartments of the lymph node (cortex, medulla, sinuses and capsule) were included.

**Gene expression profiling (GEP)**

GEP was performed by HTG according to established protocols using the Immune Response Panel (<https://www.htgmolecular.com/assets/htg/resources/C-003-05.22.R6_-_HTG-EdgeSeq_System_Brochure_.pdf>). Lysates from samples were run on the HTG EdgeSeq Processor (HTG Molecular Diagnostics, Tucson, AZ, USA) using the HTG EdgeSeq Immune Response Panel with an excess of nuclease protection probes (NPPs) complimentary to their target. S1 nuclease then removed un-hybridized probes, and RNAs leaving behind NPPs hybridized to their targets in a 1-to-1 ratio. Samples were individually barcoded using a 16-cycle PCR reaction to add adapters and molecular barcodes, individually purified using AMPure XP beads (Beckman Coulter, Brea, CA, USA) and quantitated using a KAPA Library Quantification kit (KAPA Biosystems, Wilmington, MA, USA). Libraries were sequenced on the Illumina SEQUENCER platform (Illumina, San Diego, CA, USA) for quantification. Alignment of sequenced reads was carried out by HTG using the *bowtie 2* alignment tool (<https://bowtie-bio.sourceforge.net/bowtie2/index.shtml>), which is incorporated into the proprietary *HTG EdgeSeq Parser* (version 5.0535.3138). The post-sequencing quality controls as determined by the manufacturer are met by all samples: Pearson and Spearman correlations of control samples are at least 0.85. Less than 28% of reads are attributed to positive control probes. Each library has more than 750,000 reads and finally the relative standard deviation of reads of each probe within a sample is more than 0.094. Batch effects were tested with the *RUVSeq* and *EDASeq* packages [versions 1.32.0 and 2.32.0 (1), (2)] and found to be insignificant.

Duplicate samples were merged into combined libraries. Genes with a mean log2 count <6 were discarded. The remaining genes and samples were normalized with the “median of ratios” method of the *DESeq2* package, version 1.38.0.(3) Normalized gene counts were further transformed using a variance stabilization from the *vsn* package (version 3.66.0).(4) These transformed counts were the input for the principle component analysis, the gene-count heatmaps and the gene-count boxplots.

Next, the differential gene expression p-values were estimated with the Wald-test and Bayesian shrinkage estimators were applied to the effect sizes with the *apeglm* 1.20.0 and *ashr* 2.2.54 methods.(5), (6), (7)

For gene set enrichment, five collections of the *Broad Institute*’s *Molecular Signatures Database* (<https://www.gsea-msigdb.org/gsea/msigdb>) were downloaded on 2023-07-09. The collections are the Kyoto Encyclopedia of Genes and Genomes (KEGG) gene sets, the *Reactome* gene sets, the *Wikipathways* gene sets, *the Gene Ontology: Biological Processes (*[*GO:BP*](GO:BP)*)* gene sets and the curated vaccine response (*VAX*) gene sets. The enrichment of these gene sets was tested with the *Broad Institute*’s *GSEA* algorithm,(8) implemented in the *R* package *fgsea* (version 1.24.0). Gene ranks were determined by the shrunk fold changes.

**Cell type deconvolution – cibersortx**

The approximate composition of immune cell types was inferred from the bulk-RNA data. To this end, the normalized counts were uploaded to the *cibersortx*(9) web tool on 2023-06-09. The tool was run in absolute mode with batch correction. The bulk data was mapped against the leukocyte signature matrix *LM22*. Hence only the cell types therein, i.e. within this signature, could be detected in this setup.

According to best practices in modelling compositional data,(10), (11) a centered $\log$-transform was applied to every mixture. The relative abundances between samples in disease groups of interest were modeled with a Gaussian model. The *multcomp* package, version 1.4.25,(12) was used for hypothesis tests.

*False discovery rate*

All reported p-values related to gene expression and cell types were adjusted for false discovery using the Benjamini-Hochberg method.(13).

*Software used for data analysis*

The *R* version used in the analysis is 4.2.3 (the R-Project for Statistical Computing, Vienna, Austria).

**Supplementary figure legends**

**Supplementary figure 1:**

PET Scan of patient 3 showing extensive axillary and left cervical lymphadenopathy and splenomegaly.

**Supplementary figure 2:**

Volcano plots illustrating up- and down-regulation of various genes in the respective entities.

**Supplementary figure 3:**

Volcano plot illustrating up- and down-regulation of various genes in mRNA-1237 COVID-19 vaccination-associated lymphadenopathy against all other entities.

**Supplementary figure 4:**

Heatmap of deconvoluted/approximated cell scores.

*Abbreviations:* U, unremarkable (mediastinal) lymph nodes (pale green); IM, infectious mononucleosis (gold); HLH, hemophagocytic lymphohistiocytosis (pale purple); EFPB, extrafollicular plasmablast activation (dark green); FH, follicular hyperplasia (pale brown); COVID-19 draining pulmonary lymph nodes of lethal COVID-19 (orange); mRNA-1237 COVID-19 vaccination-associated lymphadenopathy (deep purple)

**References**

1. *Normalization of RNA-seq data using factor analysis of control genes or samples.* **Risso, Davide, et al.** 2014, Nature Biotechnology, Vol. 32, pp. 896–902.

2. *GC-Content Normalization for RNA-Seq Data.* **Risso, Davide, et al.** 2011, BMC Bioinformatics, Vol. 12, p. 480.

3. *Moderated estimation of fold change and dispersion for RNA-seq data with DESeq2.* **Love, Michael I., Huber, Wolfgang and Anders, Simon.** 2014, Genome Biology, Vol. 15, p. 550.

4. *Variance stabilization applied to microarray data calibration and to the quantification of differential expression.* **Huber, Wolfgang, et al.** 2002, Bioinformatics, Vol. 18 Suppl. 1, pp. S96-S104.

5. *Heavy-tailed prior distributions for sequence count data: removing the noise and preserving large differences.* **Zhu, Anqi, Ibrahim, Joseph G. and Love, Michael I.** 2018, Bioinformatics, Vol. 35, pp. 2084-2092.

6. *False discovery rates: a new deal.* **Stephens, Matthew.** 2016, Biostatistics, Vol. 18, pp. 275-294.

7. **Stephens, Matthew, et al.** *ashr: Methods for adaptive shrinkage, using empirical bayes.* 2022. R package version 2.2-54.

8. *Gene set enrichment analysis: a knowledge-based approach for interpreting genome-wide expression profiles.* **Subramanian, Aravind, et al.** s2005, Proceedings of the National Academy of Sciences, Vol. 102, pp. 15545–15550.

9. *Determining cell type abundance and expression from bulk tissues with digital cytometry.* **Newman, Aaron M., et al.** 2019, Nature Biotechnology, Vol. 37, pp. 773–782.

10. *Benchmarking of cell type deconvolution pipelines for transcriptomics data.* **Avila Cobos, Francisco, et al.** 2020, Nature Communications, Vol. 11, p. 5650.

11. *Microbiome differential abundance methods produce different results across 38 datasets.* **Nearing, Jacob T., et al.** 2022, Nature Communications, Vol. 13, p. 342.

12. *Simultaneous inference in general parametric models.* **Hothorn, Torsten, Bretz, Frank and Westfall, Peter.** 2008, Biometrical Journal, Vol. 50, pp. 346–363.

13. *Controlling the false discovery rate: a practical and powerful approach to multiple testing.* **Benjamini, Yoav and Hochberg, Yosef.** 1995, Journal of the Royal Statistical Society, Series B (Methodological), Vol. 57, pp. 289–300.
