# Supplementary figures and images for: Case Report: Gene expression profiling of COVID-19 vaccination-related lymphadenopathies reveals evidence of a dominantly extrafollicular immune response
Source: Front Immunol. 2023 Nov 14;14:1285168. doi: 10.3389/fimmu.2023.1285168 (PMC10682704; doi:10.3389/fimmu.2023.1285168)

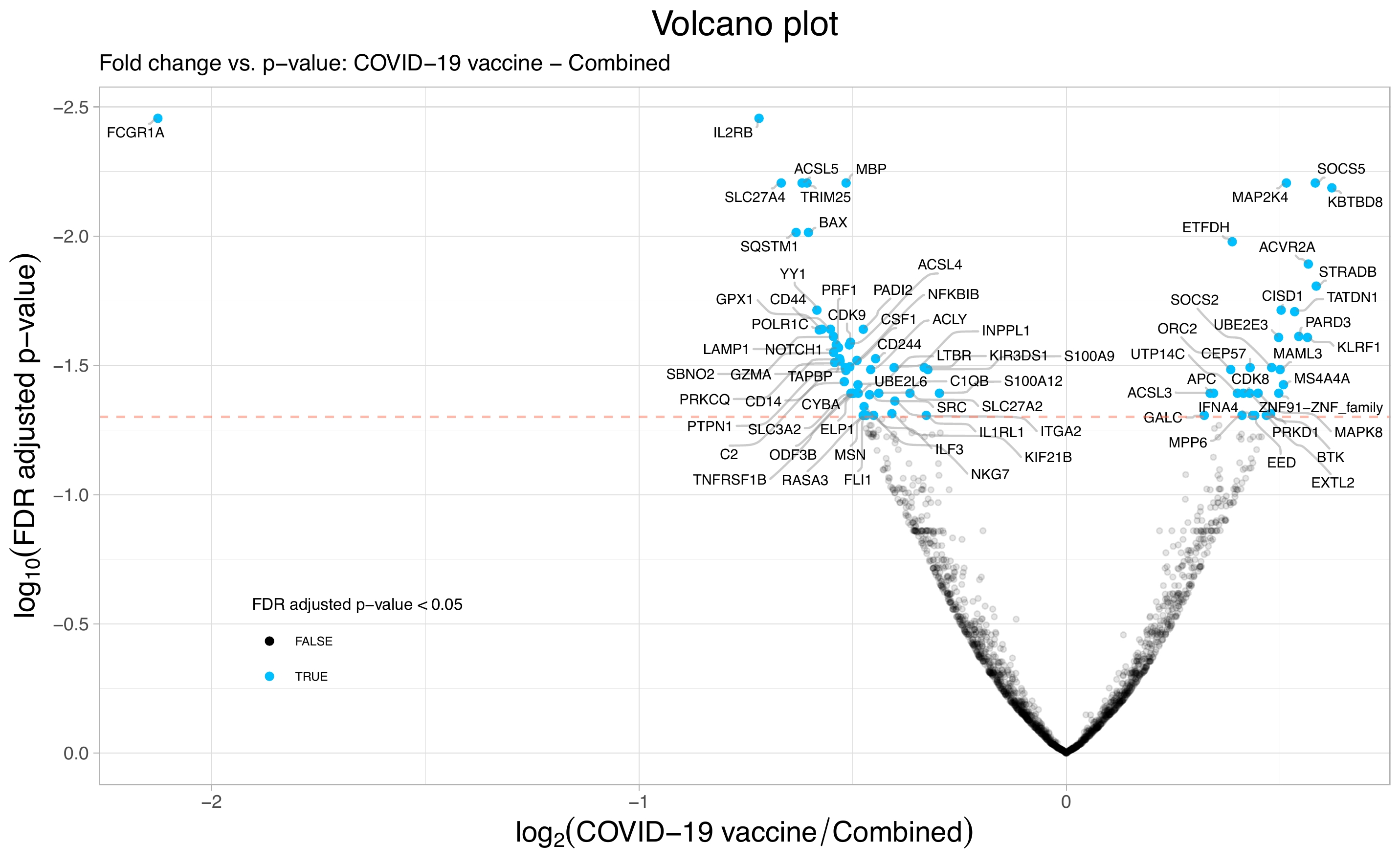

Supplement: Supplementary file 3 [file Image_1.jpeg]

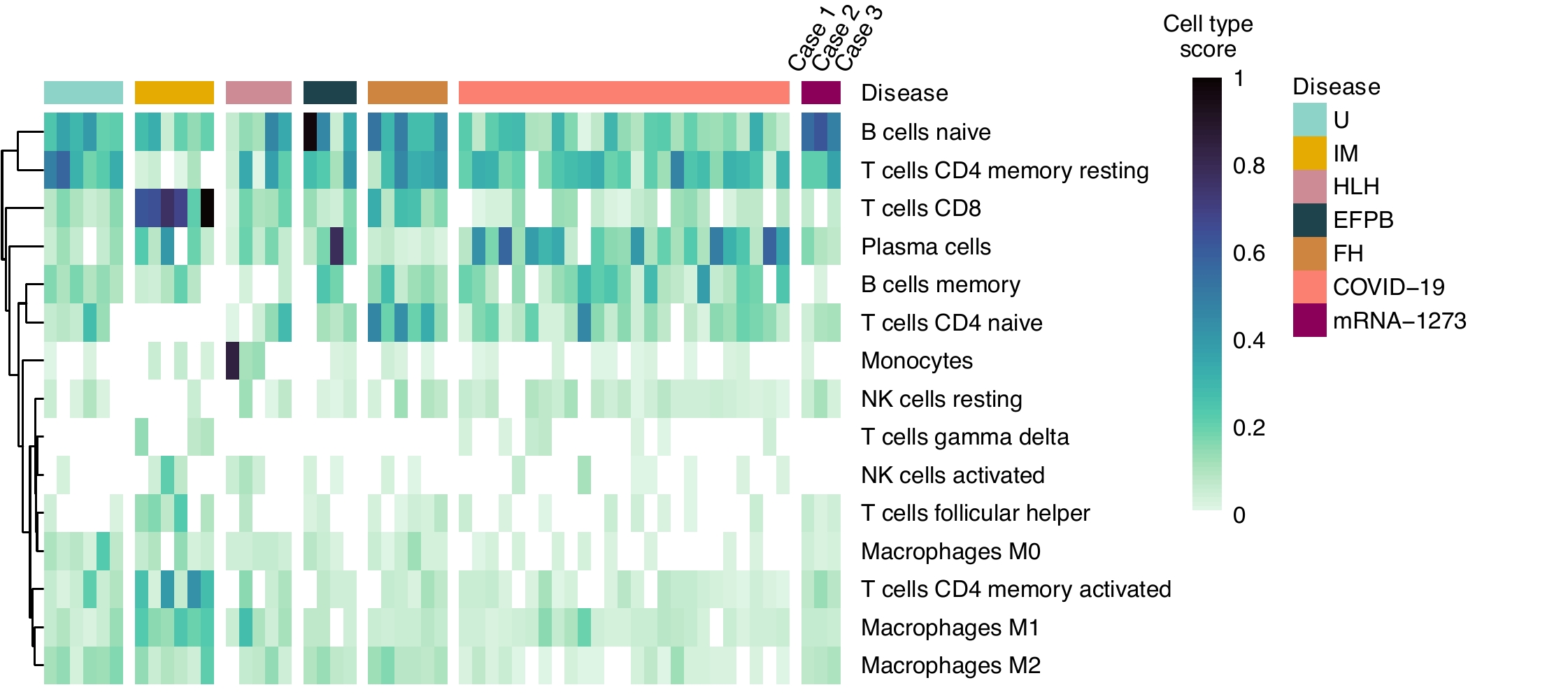

Supplement: Supplementary file 4 [file Image_2.jpeg]

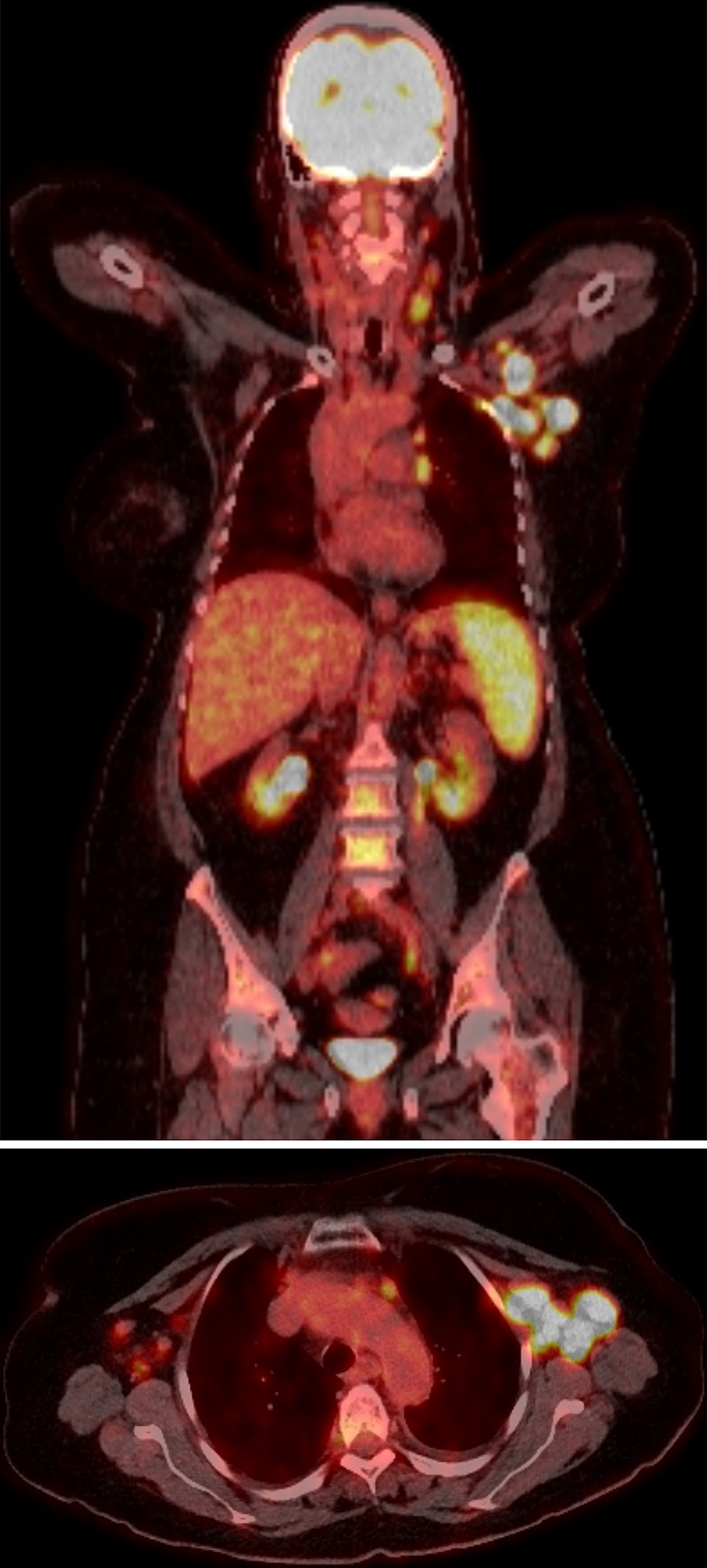

Supplement: Supplementary file 5 [file Image_3.jpeg]

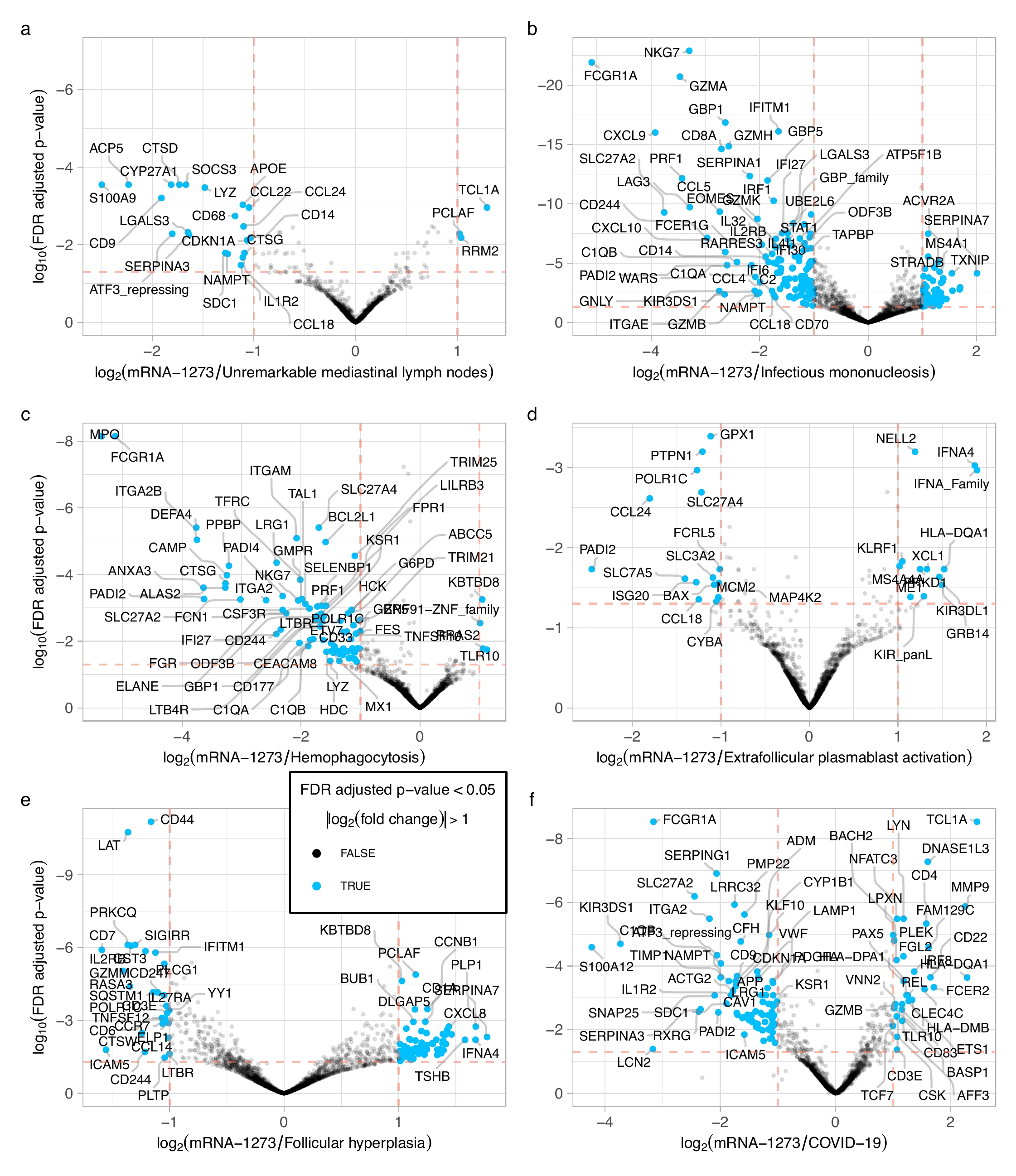

Supplement: Supplementary file 6 [file Image_4.jpeg]
